# Association Between Participation in a School-Based Structured Physical Activity Program and Standing-Height Trajectory in Thai Schoolchildren: A Quasi-Experimental Study

**DOI:** 10.3390/children13101322

**Published:** 2026-09-29

**Authors:** Apithai Bumrungpanictarworn, Chanchai Suksuwan, Oam To-aj, Santi Suksataya, Atipol Suwandee, Banalucka Tiramongkon

**Affiliations:** 1 Department of Education, Faculty of Social Sciences and Humanities, Mahidol University, Nakhon Pathom 73170, Thailand; apithai.bum@mahidol.ac.th (A.B.); oam.toa@mahidol.ac.th (O.T.-a.); 2 Faculty of Sports Science, Kasem Bundit University, Bangkok 10250, Thailand; 3 Department of Curriculum and Instruction, Faculty of Education, Prasarnmit Demonstration School (Elementary), Srinakharinwirot University, Bangkok 10110, Thailand; santis@g.swu.ac.th; 4 Graduate School, Kasem Bundit University, Bangkok 10250, Thailand; 5 Strongponlub Corp Co., Ltd., Bangkok 10260, Thailand

**Keywords:** physical activity, standing height, standing-height trajectory, schoolchildren, exercise intervention, biological maturation

## Abstract

**Highlights:**

**What are the main findings?**

- Participation in a 24-week school-based structured physical activity program was associated with a different standing-height trajectory compared with usual activity in Thai schoolchildren.
- Children participating in the program demonstrated a greater total standing-height gain over 24 weeks, with a mean between-group difference of 1.26 cm.

**What are the implications of the main findings?**

- Repeated standing-height assessment may provide additional insight into short-term standing-height trajectories during participation in a structured school-based physical activity program.
- The findings should be interpreted as preliminary associative evidence and require confirmation in larger randomized studies incorporating direct measures of biological maturation.

**Abstract:**

Background/Objectives: The relationship between structured physical activity and standing-height development in normally growing children remains insufficiently understood, as previous pediatric exercise research has predominantly focused on skeletal and fitness outcomes. This study examined the association between participation in a 24-week school-based structured physical activity program and standing-height trajectory in Thai schoolchildren. Methods: The study used a prospective non-randomized quasi-experimental design with a usual-activity comparison group and included 36 Grade 5 children aged 11–12 years. Eighteen children voluntarily participated in a structured physical activity program conducted three times per week for 24 weeks, while 18 frequency-matched children continued their usual school and daily activities. Standing height was assessed at baseline and every four weeks through Week 24. Standing-height trajectory was examined using a linear mixed-effects model adjusted for baseline standing height, age in months, and sex. Total standing-height gain and average standing-height velocity were also evaluated. Results: Standing height increased progressively in both groups. The standing-height trajectory differed significantly between the program-participation and usual-activity comparison groups. Mean total standing-height gain was 4.57 ± 1.37 cm in the program-participation group and 3.32 ± 0.92 cm in the comparison group, corresponding to a mean between-group difference of 1.26 cm (95% CI: 0.46–2.05; Hedges’ *g* = 1.05). Mean program adherence was 88.5%, and all participants in the program group met the prespecified ≥80% adherence criterion. Conclusions: Participation in the 24-week structured physical activity program was associated with a different short-term standing-height trajectory and greater total standing-height gain compared with usual activity. Because allocation was non-randomized and biological maturation was not directly assessed, these findings should be interpreted as preliminary associative evidence rather than as evidence of a causal effect on longitudinal growth.

## 1. Introduction

Growth during childhood is a multifactorial process that includes genetic, hormonal, nutritional, environmental, and behavioral influences [1,2,3]. Chronological age alone does not determine the timing or pattern of growth. Biological maturation, health, diet, rest, habitual physical activity, and other lifestyle factors influence a child’s growth and physical development. These factors become particularly important around the transition from late childhood to early adolescence, when growth velocity and pubertal timing vary substantially between individuals [4,5,6,7,8].

Physical activity supports multiple aspects of health, fitness, and development in school-aged children. Current guidance and recent systematic reviews of school-based interventions also support benefits for physical activity participation, motor competence, and physical fitness, although effects vary across intervention types and settings [9,10,11,12]. Age-appropriate programs may therefore combine movement preparation, balance and coordination, muscular fitness, and jumping and landing activities within an appropriately supervised and progressively structured format.

Weight-bearing and impact-loading activities have been studied extensively. These activities may mechanically load the skeletal system and stimulate adaptive skeletal responses [13,14]. Earlier pediatric studies and more recent systematic reviews and meta-analyses indicate that exercise can improve bone mineral content and bone mineral density during childhood and adolescence [15,16,17,18,19,20]. However, improvements in bone mass or bone strength should not be interpreted as direct evidence of accelerated longitudinal growth or greater standing height.

Exercise studies have generally reported skeletal adaptation rather than greater stature. Fuchs et al. [21] reported greater gains in bone mineral content of the femoral neck and lumbar spine after a jumping program, while standing-height changes were similar between groups. Likewise, MacKelvie et al. [22] reported bone health benefits after a school-based high-impact exercise program without a corresponding between-group difference in body-size change. These findings illustrate that skeletal adaptation and standing-height gain are distinct outcomes.

Direct evidence concerning the relationship between structured exercise and standing-height development remains limited. Wang et al. [23] reported greater height gain and changes in selected components of the growth hormone–insulin-like growth factor axis after a 24-week jumping program in children with short stature. However, the study used non-randomized group allocation, included a small exercise group, and enrolled only children with short stature; therefore, the findings cannot be generalized to normally growing schoolchildren or interpreted as causal evidence.

Interpretation of standing-height change is further complicated by biological maturation. Children aged 11–12 years may differ considerably in pubertal timing, growth velocity, and maturity status despite similar chronological ages [4,5,6,7,8]. These developmental differences may influence standing-height gain independently of participation in a structured physical activity program.

For multicomponent interventions, clear specification of content, dose, progression, supervision, permitted modifications, and delivery procedures is important for transparency and reproducibility [24]. Accordingly, the present structured physical activity program was developed as an integrated, age-appropriate school-based program rather than as a test of any single exercise component or biological mechanism.

The program incorporated movement preparation, core and neuromuscular activities, repeated jumping and landing movements, muscular-conditioning activities, and cool-down and mobility exercises within a progressively structured format. Outcomes were therefore interpreted in relation to participation in the program as a whole and not attributed to jumping, muscular conditioning, or any other component in isolation.

Repeated standing-height assessment can characterize short-term standing-height trajectory more fully than a simple pre- and post-test comparison. However, in a non-randomized quasi-experimental design, observed differences may also reflect self-selection, biological maturation, habitual physical activity, and other measured or unmeasured factors. The present study was therefore designed and interpreted as an analysis of association rather than as a test of whether the program caused an increase in longitudinal growth.

The purpose of this study was to examine the association between participation in a structured physical activity program and standing-height trajectory among 11- to 12-year-old Grade 5 students in Thailand over a 24-week period. Children who signed up for the program were examined in comparison with children in the same school and grade who continued their usual school and daily activities. Standing height was examined at the start of the study and every four weeks thereafter, at Weeks 4, 8, 12, 16, 20, and 24, resulting in seven measurements.

The primary objective was to determine the difference in standing-height trajectories between the program-participation and usual-activity comparison groups. The secondary objectives were to examine total standing-height gain and average standing-height velocity between the groups and to describe adherence to, safety, and delivery of the program.

It was expected that the children participating in the structured physical activity program would exhibit a different standing-height trajectory and greater total standing-height gain than children who engaged in their usual activities. It was assumed that the results of the study would show an association between program participation and standing-height development in Thai schoolchildren.

## 2. Materials and Methods

The study employed a prospective non-randomized quasi-experimental design with a usual-activity comparison group and repeated measurements to evaluate the association between participation in a structured physical activity program and standing-height trajectory among primary schoolchildren in Thailand. The study was conducted at a primary school in Bangkok, Thailand, over a total period of 24 weeks. The source population comprised 87 students in Grade 5 across all Grade 5 classrooms in the school. Eligibility screening was conducted for Grade 5 students regardless of the classroom to which each student belonged.

Children aged 11–12 years were the focus of this study because they are transitioning from late childhood to early adolescence, when changes in growth velocity become more pronounced and maturation timing varies substantially between individuals. Children of the same chronological age may therefore show different rates of physical growth and standing-height trajectories [4,5,6,7,8]. Focusing on one school grade and a narrow age range was intended to reduce some developmental heterogeneity within the 24-week observation period.

To be included in the study, students had to be enrolled in Grade 5 at the participating school, be aged 11–12 years, have standing height within the normal range based on the WHO growth reference [25], as determined by a height-for-age z-score (HAZ) between −2.0 and +2.0, be reported by their parent or legal guardian as being in good general health and having no known medical condition that prevented participation in regular physical activity, and provide child assent together with written informed consent from a parent or legal guardian. The use of a normal HAZ range was intended to minimize the influence of clinically atypical growth patterns on the assessment of standing-height trajectories.

Students were excluded from participation if they had a diagnosed medical or growth-related condition known to substantially affect normal growth, a medical condition that limited physical activity, a current musculoskeletal injury or condition that affected the ability to safely participate in the program, or a condition that limited the ability to safely complete standing-height measurement and/or substantially compromised completion of the study. Health information was provided by parents or legal guardians, who confirmed that their child was healthy enough to engage in regular physical activity.

An a priori sample-size calculation was performed using G*Power 3.1 (Heinrich Heine University Düsseldorf, Düsseldorf, Germany) [26] to estimate the number of participants required to detect a medium group-by-time interaction effect. A repeated-measures within–between interaction was used as an approximation for the planned longitudinal mixed-effects analysis. The effect size (Cohen’s *f*) was set at 0.25, with an α level of 0.05 and statistical power of 0.80. The assumptions included two groups, seven planned standing-height assessments, including baseline, a correlation of 0.50 among repeated measurements, and a nonsphericity correction factor of 0.50. The analysis indicated that a minimum of 28 participants was required. To allow for potential attrition and to maintain balanced group sizes, the recruitment target was conservatively set at 36 participants, with 18 participants per group.

Following eligibility screening, eligible students were classified according to whether they voluntarily participated in the structured physical activity program. Students who chose to participate formed the program-participation pool, whereas those who did not participate and continued their usual school and daily activities formed the usual-activity comparison pool. Group allocation was non-randomized. Participants were then frequency matched according to sex and chronological age in months, with 18 participants included in each group. Matching was completed before the start of the 24-week study period and was not based on subsequent standing-height measurements or participants’ responses to the program. Biological maturation was not directly assessed; therefore, matching was limited to sex and chronological age in months. No direct or surrogate indicators of biological maturation, such as Tanner stage, maturity offset or estimated peak height velocity, or skeletal age, were collected.

Because participation in the structured physical activity program was voluntary, group membership may have been influenced by self-selection. Frequency matching was used to improve comparability with respect to sex and chronological age; however, it could not account for unmeasured characteristics that may have differed between children who chose to participate and those who did not.

Standing height was measured once before commencement of the structured physical activity program and at four-week intervals during the 24-week study period. Measurements were therefore performed on seven occasions: baseline and Weeks 4, 8, 12, 16, 20, and 24. Participants were analyzed according to their study-group classification established at baseline. Because the study used a non-randomized quasi-experimental design, the focus was on the association between program participation and standing-height trajectory rather than on determining whether the program caused an increase in longitudinal growth.

The study protocol was reviewed and approved by The Committee for Research Ethics (Social Sciences), Mahidol University (Approval No.2025/030.0204). Written informed consent was obtained from parents or legal guardians, and assent was obtained from all participating children.

### 2.1. Development of the Structured Physical Activity Program

An evidence-informed, iterative process was used to establish the structured physical activity program. This focused on the existing body of pediatric physical activity research, mechanical loading, movement control, preparation, and the practical and safe means of delivering structured physical activity to school-aged children. Program development was a three-step process consisting of (i) the initial formation of the program based on existing evidence and established exercise principles, (ii) a multidisciplinary expert review and validation of the content, and (iii) piloting the program and refining it prior to implementation as part of the main study.

The program was formed as an integrated, multicomponent physical activity program as opposed to an intervention designed to evaluate the isolated effect of a single exercise component or biological mechanism. The program was constructed to include components such as activities designed for movement preparation, core and neuromuscular activities, jumping and landing activities performed on a trampoline, as well as muscular-conditioning activities and cool-down and mobility activities. These components were incorporated into the program in a structured form and progressed throughout the program period.

The primary apparatus for the repeated jumping component of the program was a trampoline. Pediatric studies have examined supervised trampoline and mini-trampoline exercise in relation to outcomes including motor performance, balance, jumping performance, and executive function [27,28,29]. These studies support the feasibility of trampoline-related activities as a supervised mode of structured physical activity for children, but they should not be interpreted as evidence that trampoline exercise directly promotes longitudinal growth or increases standing height.

The use of repeated jumping and landing was based on several pediatric studies that described the use of weight-bearing and impact-loading activities that may provide mechanical stimuli related to skeletal adaptation during the growth period [21,22,30]. While these studies focused on bone mineral accrual and other skeletal outcomes, it was determined that the studies should not be interpreted to mean that repeated jumping and landing accelerates longitudinal growth. Therefore, trampoline jumping as a form of repeated jumping and landing was introduced as a standardized, progressive component of the program, and not as an exercise intended to increase standing height.

Activities using trampolines consisted of supervised jumping and landing activities appropriate for the age group. Selection of exercises emphasized balance as well as appropriate landing technique and successful completion of the required activity. Exercise volume and the complexity of movements were adjusted based on the movement ability, quality, and tolerance of participants. Detailed descriptions of activity sequences and exercise doses are provided in Section 2.2. These descriptions include the type and duration of activities, recovery periods, and progression procedures. The beginning of each session was dedicated to controlled movement while reinforcing movement technique through dynamic movement preparation. Participants were also engaged in core and neuromuscular activities, balance and coordination, as well as muscular-conditioning activities and cool-down and mobility activities.

The intervention was assessed based on the program. Based on this assessment, any observed association with standing-height trajectory was interpreted in relation to participation in the complete structured physical activity program and not attributed to individual components such as trampoline practice, neuromuscular activities, or muscular conditioning.

The reporting of the intervention was guided by the Template for Intervention Description and Replication (TIDieR) to enhance intervention reporting and transparency. This included the intervention rationale, materials, procedures, providers, setting, dose, progression, permitted modifications, and intervention fidelity [24].

#### 2.1.1. Expert Review and Content Validation

Once the initial program was complete, the intervention protocol, exercise manual, session structure, progression framework, equipment, and safety considerations were reviewed by a multidisciplinary panel of five experts with expertise in pediatric exercise, physical activity and conditioning, biomechanics or movement science, child growth and development, and research methodology.

Experts were provided with information on the study aims, target population, rationale, proposed session and exercise structure, anticipated exercise dose, progression, safety, and program outcomes. The program manual outlined the rationale and instructions for each activity, together with the duration or volume of the activity, required recovery periods, considerations for progression and regression, required equipment, and allowable modifications.

The six domains for content validity were relevance, safety, clarity, developmental appropriateness, exercise dose, and progression. Each domain was rated on a four-point scale. An expert rating of 3 or 4 indicated agreement regarding the adequacy of the content. Experts were also allowed to provide comments and recommendations for program revisions.

The Item-Level Content Validity Index (I-CVI) was defined as the proportion of experts who rated each domain as 3 or 4. In this assessment, the domains of clarity, developmental appropriateness, exercise dose, and progression attained I-CVIs of 0.80, with agreement from four of the five experts. Relevance and safety each attained an I-CVI of 1.00. Across the six domains, I-CVI values therefore ranged from 0.80 to 1.00. The Scale-Level Content Validity Index based on the average method (S-CVI/Ave) was 0.87, defined as the mean of the six I-CVI values [31].

Although unanimous expert agreement on relevance and safety was evident, revisions were made to the domains with I-CVI values of 0.80 in preparation for the pilot study. This included consideration of the experts’ qualitative comments. The largest revisions focused on clarity of exercise instructions, developmental appropriateness of movement patterns, exercise dose, and progression criteria.

To address clarity, standardized exercise instructions and demonstrations were developed for the trampoline-based jumping and landing activities. Clarity regarding the starting position, movement sequence, landing technique, and successful completion of each activity was improved through revision of the activity manual. Standardized and consistent delivery of the program was facilitated by the inclusion of standardized instructor cues.

Developmental appropriateness was enhanced by simplifying complicated jumping patterns and providing fewer complex alternatives for movement familiarization. More complex movements could only be performed once the participants demonstrated satisfactory control of the previous movement pattern.

To allow for a more gradual introduction to activities such as repeated jumping, exercise volume was refined. During the initial phase, the volume of jumping exercises was reduced. The recovery periods between selected jumping sequences were also adjusted in order to help participants maintain movement quality and tolerance. The new criteria for progression were based on participants’ movement control and the quality of their landings in combination with their tolerance and successful completion of the previous activity or exercise. If participants did not demonstrate adequate control, completion of a predetermined number of sessions alone was not considered sufficient for progression. The revised intervention manual contained recommendations and explanations regarding trampoline positioning, supervision, permitted activity modifications, and transitions between exercises.

Following incorporation of the expert recommendations, the revised program was then implemented in the pilot study.

#### 2.1.2. Pilot Testing and Program Refinement

The revised structured physical activity program was next pilot tested with six schoolchildren who had similar age and general health characteristics to the target population. The children who participated in the pilot phase were not included in the subsequent main study.

The pilot study consisted of four weeks with three supervised sessions each week, using the same general session structure that was going to be implemented in the main study. The goal of the pilot testing was to evaluate program delivery, participants’ tolerance, appropriateness of exercise volume and progression, the practical use of the trampoline in the sessions, session length, supervision requirements, and clarity of the standardized exercise instructions. The pilot study was not designed or powered to assess changes in standing height or to assess the effectiveness of the intervention.

While conducting the pilot study, the research team observed participants’ ability to complete the activities, perform jumping and landing with adequate control, tolerate the exercise and recovery periods, and progress to the next activity level. Field notes included activity timing and sequence, transitions between program components, practical use and positioning of the trampoline, movement quality, clarity of exercise demonstrations, and supervision requirements.

We used the pilot phase to adjust the initial progression of trampoline activities, the complexity of movement, and guidance for recovery and for transitioning between the different parts of the program. We focused on getting participants familiar with the activities during the initial pilot-phase sessions before introducing more complicated jumping movements. We decided to keep less complicated jumping movements as part of the intervention for participants who needed more practice before they were ready to progress. We standardized the instructions and demonstrations for the exercises and continued to progress participants based on successful completion of the movement pattern with adequate control and tolerance.

After the four-week pilot phase, we finalized the intervention manual and began the main intervention. The final version specified the different components of the program, the activities, the exercise dose, the duration of activity and recovery periods, progression and regression criteria, permitted modifications, supervision requirements, and safety procedures. During the 24-week main intervention, we implemented the structured physical activity program as described in Section 2.2.

### 2.2. Structured Physical Activity Program and Usual-Activity Comparison Condition

Participants in the program-participation group completed a 24-week supervised structured physical activity program, consisting of three sessions per week, with each session lasting approximately 35–40 min. All intervention sessions were conducted on the premises of the participating school during the after-school period. The finalized intervention was implemented following the evidence-informed development, expert content validation, and pilot testing described in Section 2.1. All sessions were conducted by trained instructors and followed a standardized format.

#### 2.2.1. Session Structure and Exercise Content

Each session consisted of five elements: dynamic movement preparation, core and neuromuscular preparation, trampoline-based jumping and landing exercises, general muscular-conditioning exercises, and cool-down and mobility activities.

To start, each session contained approximately 5 min of dynamic movement preparation. It consisted of controlled whole-body movements as well as mobility exercises of the lower extremities involving the hip adductors, quadriceps, and hamstrings. This was followed by approximately 5 min of core and neuromuscular preparation, which included knee-up, leg-up, balance exercises, and trunk-stability exercises. Knee-up and leg-up exercises were performed 10 to 12 times for 3 sets. Short recovery periods were allowed if needed.

The main element of the session consisted of supervised trampoline-based jumping and landing activities for approximately 15 to 20 min. Activities began with bilateral vertical jumps with controlled landing. More complex jumping patterns were introduced by focusing on changes in arm position, rhythm, direction, coordination, and lower-limb movement. A standardized exercise sequence included bilateral vertical jumps, arm-assisted jumping variations, lateral leg-movement variations, and more complex coordinated jumping patterns.

Individual trampoline activities were performed in approximately 2-min bouts, with recovery periods between activity sequences. It was emphasized to participants that they should prioritize movement quality, balance, controlled take-off and landing, and consistent technique rather than maximal jump height or competitive performance. Each activity was supervised, and exercise difficulty could be reduced or recovery periods increased as needed to maintain appropriate technique and participant tolerance.

The trampoline component was followed by about 5 min of general muscular conditioning. It included bar-assisted pull-up peak holds, scissor-leg movements, and static holding exercises. Pull-up peak holds were performed for approximately 10 repetitions, scissor-leg movements for approximately 20 repetitions, and static holds for approximately 10 s. Exercise volume was reduced if there was a decrease in movement quality.

Each session was concluded with about 5 min of cool-down and mobility activities that consisted of low-intensity whole-body movements, stretching, controlled breathing, and relaxation. The standardized session structure is presented in Table 1.

#### 2.2.2. Progression Across the 24-Week Program

The 24-week intervention consisted of three phases: progressively increasing exercise volume, repetitions, and jump contacts; introducing movement complexity and coordination demands; and delineating recovery periods. Each phase was determined based on movement quality and participant tolerance in lieu of maximal exercise performance.

- Phase 1: Familiarization and Technique Development (Weeks 1–4)

Phase 1 involved participants learning basic balance and control, bilateral, and progressive jumping and landing techniques. Participants utilized the trampoline to perform lower complexity patterns in a controlled manner. Exercise volume and complexity were low, and emphasis was placed on correct performance.

- Phase 2: Progressive Volume and Movement Variation (Weeks 5–12)

In this phase, participants performed more repetitions and jump contacts. There was movement variation through changes of rhythm, arm and leg movements, and coordination requirements while maintaining a controlled landing technique. Core, neuromuscular, and muscle conditioning activities were advanced based on the participant’s ability.

- Phase 3: Consolidation and Progressive Complexity (Weeks 13–24)

In this phase, participants maintained the target volume and progressed movement variation and coordination. Trampoline movements were incorporated into more complex patterns. Movement quality, balance, and control during landing, and completion of the target dose were sustained.

Progression to a more challenging activity occurred when participants were able to successfully complete the activity, maintain control of movements, and jump and land with the correct technique. Participants were also required to tolerate the activity and recover adequately while maintaining correct technique. Those who could not properly perform all these movements were maintained at the current level or performed alternative, less complex tasks.

#### 2.2.3. Program Delivery and Instructor Standardization

The scheduled physical activity program was conducted on school grounds after regular school hours. Instructors supervised the program, which was held three times per week during the after-school period, following standardized intervention training. In preparation for the main study, instructors received standardized training on the program structure, exercise demonstration, prescribed exercise dose, movement and landing technique, participant progression and/or regression, exercise modifications, participant monitoring, and documentation.

All instructors received the same standardized training and followed the same intervention manual, including the prescribed session sequence, exercise guidelines, progression and regression criteria, permitted modifications, and documentation procedures. Intervention delivery was therefore standardized across instructors, while exercise difficulty could be maintained or reduced according to predefined movement-quality criteria and participant tolerance.

#### 2.2.4. Usual-Activity Comparison Condition

Participants in the usual-activity comparison group did not participate in the scheduled structured physical activity program during the 24-week study period. They continued their usual school and daily routines, including regularly scheduled classes, physical education, recreational play, school sports, and other customary physical activities, without any additional structured exercise prescribed by the investigators. Accordingly, the comparison condition represented usual activity rather than physical inactivity.

Habitual physical activity outside the study intervention was not systematically quantified in either group. Therefore, the frequency, duration, and intensity of extracurricular sports, leisure-time physical activity, sedentary behavior, and other daily physical activity were not included as measured exposure variables in the study.

The program-participation group differed from the comparison group by the addition of three supervised structured physical activity sessions per week. Both groups underwent the same scheduled standing-height assessments, as described in Section 2.3.

### 2.3. Standing-Height Assessment and Study Outcomes

Standing height was measured at baseline and at Weeks 4, 8, 12, 16, 20, and 24. All measurements were conducted at the participating school during the same scheduled after-school assessment period. For participants in the program-participation group, measurements were completed prior to the scheduled exercise session.

A Nagata stadiometer (BW-1410H; Yung Tai Scale Co., Ltd., Tainan, Taiwan) was used for all assessments following standardized measurement procedures. All measurements were performed by the same assessor throughout the study. Participants were measured barefoot and in light clothing, with their arms relaxed at their sides and their heads positioned in the Frankfort horizontal plane. Height was recorded to the nearest 0.1 cm. Each participant was measured twice at each assessment, and the mean of the two measurements was used for analysis.

The primary endpoint was the 24-week standing-height trajectory. The secondary endpoints were standing-height gain and standing-height velocity. Standing-height velocity was determined by the change in standing height from baseline to the end of the study, with the 24-week period expressed in years, and was reported in cm/year. Changes in standing height over the 24 weeks were also summarized between consecutive 4-week intervals.

Changes in measured standing height were interpreted as changes in measured stature over time rather than as a direct measure of longitudinal bone growth.

### 2.4. Adherence, Intervention Fidelity, and Safety Monitoring

Attendance was recorded at each intervention session using participant sign-in records. Adherence was calculated as the number of sessions attended divided by the total number of scheduled sessions and expressed as a percentage. Participants attending at least 80% of the scheduled sessions were classified as adherent.

Intervention fidelity was monitored using standardized session records based on the intervention manual. Instructors documented completion of each planned session component, any modification to exercise progression or regression, and the reason for any deviation from the prescribed program. These records were used to monitor consistency of program delivery throughout the 24-week intervention.

Instructors were responsible for monitoring participant safety during all session activities. Any exercise-related symptoms or adverse events were documented. Activities were modified or discontinued if participants reported pain or discomfort, fatigue, loss of control, injury or potential injury, or any other safety concern.

### 2.5. Statistical Analysis

Statistical analyses were performed with IBM SPSS Statistics for Windows, Version 22.0 (IBM Corp., Armonk, NY, USA). Continuous variables were reported as either the mean with the standard deviation or the median and interquartile range, and categorical variables as counts and percentages.

Standing-height trajectories were analyzed using a linear mixed-effects model. The six post-baseline measurements at Weeks 4, 8, 12, 16, 20, and 24 were treated as repeated outcomes. Time was treated as a categorical fixed effect, with group and the group × time interaction also included as fixed effects. Baseline standing height, age in months, and sex were included as covariates. Participant-specific random intercepts and random linear slopes for time were included as random effects to account for within-participant correlation across repeated measurements. Model parameters were estimated using restricted maximum likelihood. The group × time interaction was the primary effect of interest. Estimated marginal means and corresponding 95% confidence intervals were reported. Model diagnostics were examined to assess the adequacy of the fitted model, and no major violations of the model assumptions were identified.

The mean change in standing height from baseline to Week 24 was compared between groups using Welch’s independent-samples *t*-test. The mean between-group difference was reported with its corresponding 95% confidence interval, and Hedges’ *g* was calculated as a standardized effect size. Average standing-height velocity and changes in standing height between consecutive four-week assessments were summarized descriptively. Program adherence was also analyzed descriptively. All standing-height data were checked for completeness and accuracy before analysis. Complete data were available for all participants at all scheduled assessment points; therefore, no imputation was required. A two-sided *p* value < 0.05 was considered statistically significant.

## 3. Results

### 3.1. Participant Characteristics

Thirty-six children were analyzed, consisting of two groups of eighteen children, one group involved in program participation and one group acting as the usual-activity comparison group. There were no missing data for standing-height measurements for any participant at baseline or at the 4-, 8-, 12-, 16-, 20-, and 24-week time points of the study.

The average age of the program-participation group was 136.06 months (SD 2.82 months) and 136.11 months (SD 2.35 months) for the usual-activity comparison group. There were nine boys (50.0%) and nine girls (50.0%) in each group. The average baseline standing-height measurement for the program-participation group was 142.21 cm (SD 5.15 cm), and for the usual-activity comparison group it was 140.57 cm (SD 5.15 cm). Baseline characteristics are summarized in Table 2.

### 3.2. Program Adherence and Implementation

All 72 intervention sessions were completed during the 24-week program. Participants in the program-participation group attended an average of 63.72 sessions (SD 3.97 sessions), resulting in an average adherence of 88.50% (SD 5.51%), with individual adherence ranging from 80.56% to 95.83%.

The prespecified adherence criterion of attending at least 80% of the sessions was met by all 18 participants (100%). Throughout the 24-week intervention, the structured session design and sequence were maintained without major modifications to the intervention protocol. No exercise-related adverse events or injuries were recorded during the 24-week program, and no intervention sessions were discontinued because of safety concerns.

### 3.3. Standing-Height Trajectory Across the 24-Week Study Period

During the 24-week study period, standing height was observed to increase in both study groups. In the program-participation group, the mean standing height increased from 142.21 ± 5.15 cm at baseline to 146.78 ± 5.79 cm at Week 24. In the usual-activity comparison group, the mean standing height increased from 140.57 ± 5.15 cm at baseline to 143.89 ± 5.38 cm at Week 24 (Table 3).

In the linear mixed-effects model adjusted for baseline standing height, age in months, and sex, the group × time interaction was statistically significant, F(5, 98.83) = 2.49, *p* = 0.036, indicating that the pattern of change in standing height differed between the program-participation group and the usual-activity comparison group.

The adjusted between-group difference was 0.27 cm (95% CI −0.02 to 0.56) at Week 4 and increased to 1.27 cm (95% CI 0.45 to 2.09) at Week 24 (Table 4).

### 3.4. Total Standing-Height Gain and Average Standing-Height Velocity

During the 24-week study period, the program-participation group had a mean standing-height gain of 4.57 ± 1.37 cm, compared with 3.32 ± 0.92 cm in the usual-activity comparison group.

The mean between-group difference in total standing-height gain was 1.26 cm (95% CI 0.46–2.05). Welch’s independent-samples *t*-test indicated that this difference was statistically significant, *t*(29.73) = 3.23, *p* = 0.003, with Hedges’ *g* = 1.05.

The mean annualized standing-height velocity was 9.91 ± 2.97 cm/year in the program-participation group and 7.19 ± 1.99 cm/year in the usual-activity comparison group. Standing-height velocity was reported descriptively because it represents an annualized transformation of standing-height gain over the same 24-week observation period. These outcomes are summarized in Table 5.

### 3.5. Four-Week Changes in Standing Height

Changes in standing height across the four-week intervals were analyzed descriptively. For the program-participation group, mean changes in standing height in each of the four-week intervals ranged from 0.69 to 0.86 cm. For the usual-activity comparison group, mean changes in standing height ranged from 0.46 to 0.64 cm. The interval-specific changes are presented in Table 6.

These interval-specific changes were considered exploratory and were therefore reported descriptively without separate inferential testing.

## 4. Discussion

This study examined whether participation in a 24-week structured physical activity program was associated with standing-height trajectory in Thai schoolchildren. Standing height increased in both groups, as expected during normal childhood growth, but the pattern of change differed significantly between the program-participation and usual-activity comparison groups. The program group showed a greater total standing-height gain, with a mean between-group difference of 1.26 cm. Because the study used a non-randomized quasi-experimental design and biological maturation was not directly assessed, these findings should be interpreted as associative rather than causal. The clinical significance of the observed difference also remains uncertain given the relatively short observation period and normal variation in childhood growth.

Previous exercise studies reinforce the distinction between skeletal adaptation and standing-height gain. Fuchs et al. [21] reported greater gains in lumbar-spine and femoral-neck bone mineral content following a high-impact jumping program in prepubescent children, without a corresponding difference in standing height. Similarly, MacKelvie et al. [22,32] reported improvements in bone mineral content and structural properties following school-based high-impact exercise, while body-size changes did not differ significantly between groups. Longitudinal evidence also suggests that greater physical activity does not necessarily translate into greater statural growth. Beunen et al. [33] found no significant relationship between physical activity levels and somatic growth, skeletal maturation, or the timing of peak height velocity, while Malina [34] reported that regular physical activity and sports training did not consistently influence attained stature or growth rate. Collectively, these findings indicate that skeletal benefits from exercise should not be interpreted as evidence of enhanced longitudinal growth.

More recent studies have examined height-related outcomes more directly. Wang et al. [23] reported greater height gain and increased femoral-neck bone mineral density following a 24-week progressive jumping program in children with short stature, with similar findings reported in another 24-week study [35]. However, these populations differ importantly from that of the present study. Children with short stature may differ from normally growing children in maturational status, endocrine characteristics, nutritional status, and potential for catch-up growth. Findings from short-stature populations therefore cannot be directly generalized to schoolchildren with standing height within the normal range for age. The present study extends this area of research by examining repeated standing-height changes in normally growing children.

The characteristics of the intervention may also be relevant to the observed pattern. Participants completed three supervised sessions per week for 24 weeks, incorporating progressive jumping and landing activities, neuromuscular preparation, muscular-conditioning exercises, and movement-control activities. Mean adherence was 88.5%, and all participants met the prespecified minimum adherence criterion, indicating consistent exposure to the planned program. Because the intervention was multicomponent, however, the contribution of any single activity cannot be determined. The observed differences should therefore be interpreted in relation to participation in the structured program as a whole rather than attributed specifically to trampoline-based jumping or any other individual component.

Mechanical loading provides a possible biological context for interpreting the findings, but mechanistic conclusions should remain cautious. The growth plate functions within a complex mechanical, endocrine, and developmental environment, and previous reviews suggest that mechanical loading may influence growth-plate physiology [36]. However, the present study did not assess growth-plate morphology, skeletal age, bone mineral density, growth hormone, or IGF-1. Accordingly, the observed difference in standing height should not be interpreted as evidence of growth-plate stimulation or activation of the growth hormone system.

Several limitations should be considered. First, the non-randomized design and voluntary participation introduce the possibility of selection bias and residual confounding. Children who chose to participate may have differed from those who did not in unmeasured characteristics, including prior sport participation, lifestyle behaviors, family support, or other health-related factors. Although participants were frequency matched by sex and age in months and the longitudinal model adjusted for baseline standing height, age, and sex, these procedures cannot fully account for unmeasured between-group differences. Habitual physical activity outside the intervention was also not systematically quantified, and the relatively small sample from a single school may limit statistical precision and generalizability.

Second, biological maturation and several other potentially relevant growth-related variables—including nutritional intake, sleep, body weight, endocrine markers, parental height, pubertal status, and familial growth characteristics—were not directly assessed. These factors may have contributed to the observed standing-height trajectories. In addition, standing height is an anthropometric measure of stature and cannot determine longitudinal bone elongation or growth-plate activity. The 24-week observation period also represents only a short interval of childhood growth and cannot establish whether the observed between-group difference would persist over a longer period.

Despite these limitations, the study has several strengths. The structured program was implemented in a real school setting, all scheduled sessions were delivered, and participant adherence was high. The use of a usual-activity comparison group also reflected typical school-day conditions. Importantly, standing height was assessed at seven time points over 24 weeks, allowing standing-height trajectory to be examined longitudinally rather than relying only on a baseline-to-endpoint comparison. Repeated standing-height trajectories remain under-researched among normally growing children, and the present study provides preliminary evidence that may inform future investigations.

Future studies should include larger and more diverse samples, randomized or cluster-randomized controlled designs, direct assessment of biological maturation, and longer follow-up. Repeated height measurements should ideally be examined alongside habitual physical activity, nutritional factors, skeletal measures, and relevant biological growth markers. Such studies will be needed to determine whether differences in standing-height trajectory are associated with structured physical activity itself or primarily reflect normal variation in childhood growth.

## 5. Conclusions

Participation in a 24-week school-based structured physical activity program was associated with a different standing-height trajectory and greater total standing-height gain compared with usual activity among Thai schoolchildren. Because the study used a non-randomized quasi-experimental design and did not directly assess biological maturation or several other growth-related factors, the between-group differences cannot be attributed to the program itself. The findings should therefore be interpreted as preliminary associative evidence regarding short-term standing-height trajectory and standing-height gain, not as evidence of enhanced or accelerated longitudinal growth. Larger randomized studies with longer follow-up and assessment of maturation, habitual physical activity, nutrition, and relevant skeletal and biological growth indicators are needed to clarify this relationship.

## Figures and Tables

**Table 1:** Structure and Content of the 24-Week Structured Physical Activity Program.

Program Component	Approximate Duration	Exercise Content
Dynamic movement preparation	5 min	Controlled whole-body movements and lower-limb mobility involving the adductors, quadriceps, and hamstrings
Core and neuromuscular preparation	5 min	Knee-up, leg-up, balance, trunk-stability, and movement-control activities
Trampoline-based jumping and landing	15–20 min	Bilateral vertical jumps, arm-assisted variations, lateral leg-movement variations, and progressively coordinated jumping patterns
General muscular conditioning	5 min	Bar-assisted pull-up peak holds, scissor-leg movements, and static holding exercises
Cool-down and mobility	5 min	Low-intensity movement, stretching, controlled breathing, and relaxation
Total	35–40 min	

**Table 2:** Baseline characteristics of the study participants.

Characteristic	Program-Participation Group (*n* = 18)	Usual-Activity Comparison Group (*n* = 18)	Total (N = 36)
**Age, months, mean ± SD**	136.06 ± 2.82	136.11 ± 2.35	136.08 ± 2.56
**Boys, *n* (%)**	9 (50.0)	9 (50.0)	18 (50.0)
**Girls, *n* (%)**	9 (50.0)	9 (50.0)	18 (50.0)
**Baseline standing height, cm, mean ± SD**	142.21 ± 5.15	140.57 ± 5.15	141.39 ± 5.14

**Table 3:** Observed standing height across assessment occasions.

Assessment	Program-Participation Group, Mean ± SD (cm)	Usual-Activity Comparison Group, Mean ± SD (cm)
**Baseline**	142.21 ± 5.15	140.57 ± 5.15
**Week 4**	142.95 ± 5.11	141.06 ± 5.15
**Week 8**	143.81 ± 5.29	141.52 ± 5.13
**Week 12**	144.55 ± 5.36	142.13 ± 5.29
**Week 16**	145.24 ± 5.45	142.72 ± 5.39
**Week 20**	146.00 ± 5.59	143.36 ± 5.38
**Week 24**	146.78 ± 5.79	143.89 ± 5.38

**Table 4:** Estimated marginal means for standing height across the 24-week study period.

Assessment	Program-Participation Group, Adjusted Mean (95% CI), cm	Usual-Activity Comparison Group, Adjusted Mean (95% CI), cm	Adjusted Difference (95% CI), cm
**Week 4**	142.14 (141.94–142.34)	141.87 (141.67–142.07)	0.27 (−0.02–0.56)
**Week 8**	143.00 (142.74–143.26)	142.33 (142.07–142.59)	0.67 (0.31–1.04)
**Week 12**	143.74 (143.41–144.07)	142.94 (142.61–143.27)	0.80 (0.33–1.26)
**Week 16**	144.44 (144.03–144.84)	143.53 (143.12–143.94)	0.90 (0.33–1.48)
**Week 20**	145.19 (144.70–145.68)	144.17 (143.68–144.66)	1.02 (0.32–1.72)
**Week 24**	145.97 (145.39–146.55)	144.70 (144.12–145.28)	1.27 (0.45–2.09)

Note: Estimated marginal means were adjusted for baseline standing height, age in months, and sex.

**Table 5:** Total standing-height gain and average standing-height velocity over 24 weeks.

Outcome	Program-Participation Group	Usual-Activity Comparison Group	Between-Group Difference
**Total standing-height gain,** **cm, mean ± SD**	4.57 ± 1.37	3.32 ± 0.92	1.26 (95% CI 0.46–2.05)
**Average standing-height velocity, cm/year, mean ± SD**	9.91 ± 2.97	7.19 ± 1.99	—

Note: The between-group difference in total standing-height gain was assessed using Welch’s independent-samples *t*-test: *t*(29.73) = 3.23, *p* = 0.003; Hedges’ *g* = 1.05. The between-group difference was calculated using unrounded values.

**Table 6:** Changes in standing height between consecutive 4-week assessments.

Interval	Program-Participation Group, Mean ± SD (cm)	Usual-Activity Comparison Group, Mean ± SD (cm)
**Baseline–Week 4**	0.74 ± 0.35	0.49 ± 0.27
**Week 4–Week 8**	0.86 ± 0.34	0.46 ± 0.32
**Week 8–Week 12**	0.74 ± 0.31	0.61 ± 0.41
**Week 12–Week 16**	0.69 ± 0.29	0.59 ± 0.34
**Week 16–Week 20**	0.76 ± 0.36	0.64 ± 0.32
**Week 20–Week 24**	0.78 ± 0.30	0.53 ± 0.28

## Data Availability

The data supporting the findings of this study are not publicly available due to privacy and ethical considerations related to research involving children. The data may be available from the corresponding author upon reasonable request, subject to applicable ethical and institutional requirements.

## Abbreviations

The following abbreviations are used in this manuscript:

BMC	Bone mineral content
CI	Confidence interval
CVI	Content Validity Index
HAZ	Height-for-age z-score
I-CVI	Item-Level Content Validity Index
IGF-1	Insulin-like growth factor 1
S-CVI/Ave	Scale-Level Content Validity Index based on the average method
SD	Standard deviation
TIDieR	Template for Intervention Description and Replication
